# Legume Protein Consumption and the Prevalence of Legume Sensitization

**DOI:** 10.3390/nu10101545

**Published:** 2018-10-19

**Authors:** Mark Smits, Thuy-My Le, Paco Welsing, Geert Houben, André Knulst, Kitty Verhoeckx

**Affiliations:** 1 Department of Dermatology/Allergology, University Medical Center Utrecht, 3584 CX Utrecht, The Netherlands; T.T.M.Le-2@umcutrecht.nl (T.-M.L.); P.M.J.Welsing@umcutrecht.nl (P.W.); geert.houben@tno.nl (G.H.); A.C.Knulst@UmcUtrecht.nl (A.K.); kitty.verhoeckx@tno.nl (K.V.); 2 TNO, 3704 HE Zeist, The Netherlands; 3 Laboratory of Translational Immunology, University Medical Center Utrecht, Utrecht University, 3584 CX Utrecht, The Netherlands

**Keywords:** legumes, consumption, prevalence, prediction, sensitization, food hypersensitivity, food allergy, peanut

## Abstract

Sensitization and allergy to legumes can be influenced by different factors, such as exposure, geographical background, and food processing. Sensitization and the allergic response to legumes differs considerably, however, the reason behind this is not yet fully understood. The aim of this study is to investigate if there is a correlation between legume protein consumption and the prevalence of legume sensitization. Furthermore, the association between sensitization to specific peanut allergens and their concentration in peanut is investigated. Legume sensitization data (peanut, soybean, lupin, lentil, and pea) from studies were analyzed in relation to consumption data obtained from national food consumption surveys using the European Food Safety Authority (EFSA), Global Environment Monitoring System (GEMS), and What We Eat in America—Food Commodity Intake Database (WWEIA-FCID) databases. Data were stratified for children <4 years, children 4–18 years, and adults. Sufficient data were available for peanut to allow for statistical analysis. Analysis of all age groups together resulted in a low correlation between peanut sensitization and relative peanut consumption (*r =* 0.407), absolute peanut consumption (*r =* 0.468), and percentage of peanut consumers (*r =* 0.243). No correlation was found between relative concentrations of Ara h 1, 2, 3, 6, 7, and 8 in peanut and sensitization to these peanut allergens. The results indicate that the amount of consumption only plays a minor role in the prevalence of sensitization to peanut. Other factors, such as the intrinsic properties of the different proteins, processing, matrix, frequency, timing and route of exposure, and patient factors might play a more substantial role in the prevalence of peanut sensitization.

## 1. Introduction

The prevalence of food allergy is thought to be on the rise. In contrast, evidence has also been published that the prevalence of food sensitization and food allergy has plateaued at an all-time high [1,2]. Studies investigating the prevalence of food allergy at multiple time points are scarce. In one of these studies, Hu et al. found that food allergy increased significantly from 3.5% in 1999 to 7.7% in 2009 in 0–2-year-old Chinese children [3]. Furthermore, Sicherer et al. showed that the prevalence of self-reported peanut allergy increased from 0.4% in 1997 to 1.4% in 2008 and for tree nut allergy from 0.2% in 1997 to 1.1% in 2008 [4]. Studies investigating the prevalence of food sensitization point in the same direction. Although sensitization to a food does not invariably induce food allergy, it is an essential prerequisite and an indicator of the prevalence of food allergy. In the study of Hu et al., food sensitization increased from 9.9% in 1999 to 18% in 2009. In addition, peanut sensitization increased threefold in the United Kingdom from 1.1% in 1989 to 3.3% in 1994 to 1996 [5]. Peanut sensitization and allergy was also found to be higher, although not significantly, in the study of Venter et al. in two birth cohorts born 12 years apart [6].

Additionally, differences in the prevalence of food sensitization between geographical locations have been described. For example, a study in 1-year-old children in China in 2009 showed a high prevalence (11.3%) of egg-sensitized children, compared to a low prevalence (0.4%) of peanut-sensitized children [7]. A similar study in 1-year-old children in Australia in 2009 reported a similar prevalence of egg sensitization (11.7%), but a 16-fold higher prevalence of peanut sensitization (6.4%) compared to the Chinese study [8]. Differences in sensitization rates were also seen in two neighboring countries, Finland and Russia. In this case, 12.2% of Finnish children were sensitized to peanut compared to 1.2% of Russian children, and sensitization to soybean was 4.1% in Finland compared to 0% in Russia [9]. A possible explanation could be differences in legume consumption (e.g., amount and frequency) or dietary habits (e.g., processing). Amount, timing, and frequency of legume consumption could influence the prevalence of legume sensitization as well. However, the relation between legume consumption and the prevalence of legume sensitization has currently not been investigated.

Legumes are implicated in many cases of food allergy. Recently, Sasaki et al. reported food allergy in 4.5% of Australian adolescents and a high frequency of peanut (2.7%) and soybean (0.1%) allergy [10]. Peanut and soybean are part of the *Fabaceae* or legume family, which in addition includes lentils, lupin, peas, and beans. Several legume allergens have been well-characterized and are listed in the Supplementary Materials (Table S1) [11]. For example, multiple allergens have been identified in peanut (Ara h 1–17). The amounts of these allergenic proteins in peanut differ considerably. Peanut mainly consists of the seed storage proteins Ara h 1 (7S globulin), Ara h 2 (2S albumin), Ara h 3 (11S glycinin), and Ara h 6 (2S albumin) [12]. Ara h 2 and Ara h 6 sensitization is found to be associated with severe reactions [13]. Sensitization to allergenic proteins present in lower amounts such as Ara h 8 (homologue to Bet v 1) and 9 (a non-specific lipid transfer protein) appears to be clinically less relevant compared to sensitization to seed storage proteins [14,15]. Sensitization to soybean seed storage proteins (Gly m 5 and Gly m 6) was associated with severe allergic reactions [16,17]. Cross-reactivity between legumes is often seen, however, clinical reactivity to multiple legumes is reported to be low [18]. Vicilin and convicilin from pea were identified as major allergens, and cross-reactivity with the major allergen from lentil (Len c 1) occurred in all 18 pea allergic patients in Spain [19,20]. Additionally, in peanut-allergic patients, co-sensitization to lupine (82%), pea (55%), and soybean (87%) is often seen [21]. Peanut specific immunoglobulin E (IgE) was inhibited by soybean (26%) extract, thus indicating possible cross-reactivity [22]. Klemans et al. concluded that Ara h 2 had the best predictive value for diagnosing peanut allergy in adults [23]. Since Ara h 2 is a dominant protein in peanut, this could indicate that a high concentration is associated with a higher prevalence of sensitization for that protein. However, this possible association has not yet been investigated.

In this study, the prevalence of legume sensitization in continental regions as reported in literature was combined with reported consumption data from acclaimed consumption surveys from the European Food Safety Authority (EFSA), the Global Environment Monitoring System (GEMS), and the What We Eat in America—Food Commodity Intake Database (WWEIA-FCID) databases. By associating consumption and sensitization data, we aimed to investigate if the amount of consumption of legumes influences the prevalence of legume sensitization. Furthermore, the association between sensitization to specific peanut allergens and their concentrations in peanut is investigated.

## 2. Materials and Methods 

### 2.1. Literature Search

Articles investigating the prevalence of sensitization to legumes were retrieved from electronic bibliographic databases (Scopus, Web of Science, PubMed, and EMbase). The search queries for the electronic bibliographic databases are listed in the Supplementary Materials (Table S2). To investigate if sensitization to peanut allergens, such as Ara h 2 and 6, were correlated to the content of these allergens in peanut, another literature search was conducted. All articles fulfilling the search criteria (“Ara h” and “Sensitization”) in the electronic bibliographic databases were evaluated fully. The search queries for this study are listed in the Supplementary Materials (Table S3). The results of the search queries were uploaded in Datawarehouse Infrastructure for Applications, Models and Ontologies towards Novel Design and Safety 2 (DIAMONDS2, TNO, Zeist, The Netherlands). DIAMONDS2 was used to evaluate and select the articles fulfilling the inclusion and exclusion criteria.

### 2.2. Inclusion and Exclusion Criteria

Regarding the prevalence of sensitization to legumes, cohort studies, cross-sectional studies, longitudinal studies, case-control studies, and routine healthcare studies were included for review. Reviews, discussion papers, case studies, and animal studies were excluded. Publications in English, German, and Dutch were included; there was no restriction in year of publication. Articles investigating the prevalence of legume sensitization in the general population were included. Sensitization was defined as a positive skin prick test (wheal size of >3 mm diameter) or specific immunoglobulin E (sIgE) antibodies in blood (>0.35 kU/L on ImmunoCAP (ThermoFisher, Uppsala, Sweden) or >0.3 ISAC Standardized Units on ImmunoCAP ISAC 112 (ThermoFisher, Uppsala, Sweden)).

Regarding the prevalence of sensitization to peanut allergens, articles investigating the prevalence or frequency for specific peanut protein sensitization (Ara h 1–17) were selected. Articles that evaluated specific peanut protein sensitization in patients with either a convincing history of peanut allergic reactions in combination with a positive laboratory test (skin prick test or sIgE testing in blood) or an oral food challenge-confirmed peanut allergy were included.

### 2.3. Consumption and Sensitization Data Collection

Consumption data on legumes were retrieved from the EFSA (accessed on 2 November 2017), GEMS/Food consumption (accessed on 20 December 2017), and WWEIA-FCID (accessed on 2 December 2018) consumption databases. The EFSA and GEMS/Food consumption databases contain national dietary surveys, and the WWEIA-FCID consumption database reports food consumption in the United States. The consumption value for a study was based on the mean age of the study population (0–1 year for infants; 1–4 years for toddlers; 4–10 years for children; 10–18 years for adolescents; and >18 years for adults) and, if possible, was close to the year of publication of the study. Chronic food consumption statistics in the consumption surveys were reported in gram per day (absolute) and gram/kg bodyweight (bw) per day (relative). The mean percentage of peanut consumers was calculated by averaging the peanut and peanut butter consumer percentages. Relative and absolute consumption data and the percentage of consumers were used to investigate possible correlation between consumption behavior and legume sensitization.

Using a custom evaluation sheet, the reported prevalence of legume sensitization, the diagnostic test used, the age and size of the study population, and the country in which the study was conducted was noted. An overview table with study information can be found in the Supplementary Materials (Table S4). The consumption and percentage value in the corresponding age group was retrieved from the consumption survey. When no corresponding consumption data was available for the country, the study was excluded. Studies were stratified for age of the study population in children <4 years, children 4–18 years, and adults. For peanut consumption, we used the sum of peanut and peanut butter consumption from the EFSA database. 

The relative and absolute consumption values were corrected for the percentage of protein present in the food product. Percentage of protein in the product was investigated using the United States Department of Agriculture (USDA) Food Composition Database (accessed on 2 February 2018). Peanut, peanut butter, and peanut oil were the major peanut products investigated in the consumption surveys. Peanut and peanut butter consist of 25% protein according to the USDA Food Composition Databases. Peanut oil does not contain peanut protein and was therefore not included in the total peanut protein consumption. In the case of soybean consumption, inconsistencies between consumption surveys were found. Soybean consumption was calculated using the soybean products as reported in the national consumption survey, making the assumption that when products are not listed that these products are not eaten.

The correlation between total legume protein consumption and the prevalence of sensitization in a country was investigated.

### 2.4. Statistical Analysis

The correlation (*r* value) between legume consumption, percentage legume consumers, and legume sensitization data was examined using weighted least squares (WLS) linear regression analysis (SPSS version 21, IBM, Armonk, NY, USA). The size of the *r* value indicates the strength of the relationship between two variables and is considered to be negligible (0.0–0.3), low (0.3–0.5), moderate (0.5–0.7), strong (0.7–0.9), or very strong (0.9–1.0) [24].

The correlation between the frequency of specific peanut protein sensitization (Ara h 1, 2, 3, 6, 7, and 8) and the specific amount of this protein in peanut was analyzed using Spearman’s rank correlation coefficient (SPSS version 21, IBM, Armonk, NY, USA). Mean sensitization values per specific protein were calculated based on the sensitization frequency reported in the articles. The mean amount of the specific peanut protein allergens was based on the reported values in literature (Table S5). In both studies, a *p* value of <0.05 was considered statistically significant.

## 3. Results

### 3.1. Legume Sensitization Literature Search

**Figure 1:**
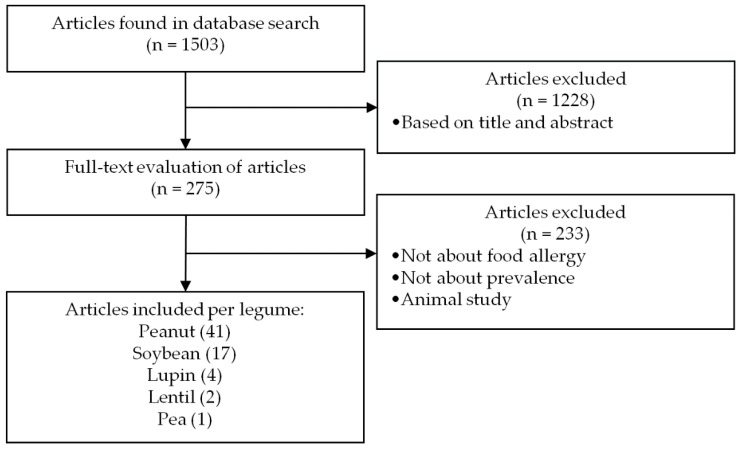
Flow diagram of the literature search approach.

The search results are summarized in the flowchart of Figure 1. In total, 1503 unique articles were retrieved from four electronic bibliographic databases and evaluated. Of the 1503 retrieved articles, 1228 were excluded based on language, title, and abstract screening. After full-text analysis of the remaining 275 articles, 233 were excluded based on study type, study population, or scope of the publication. Forty-two articles met the inclusion criteria and were subsequently included in our analysis. Of the selected articles, 41 articles investigated peanut sensitization, 17 soybean sensitization, 4 lupin sensitization, 2 lentil sensitization, and 1 pea sensitization in the general population. The majority of the studies selected were performed in Europe or North America; limited sensitization prevalence data were available for Asia, Africa, and South America.

### 3.2. Peanut Allergen Sensitization Literature Search

In the case of sensitization to specific peanut allergens, the literature search resulted in 269 articles, which were fully evaluated. Twenty-one articles were selected that investigated peanut allergen sensitization in patients with either a convincing history of peanut allergic reactions in combination with a positive laboratory test (skin prick test or sIgE testing in blood) or an oral food challenge-confirmed peanut allergy.

### 3.3. Peanut Protein Consumption and Sensitization

**Figure 2:**
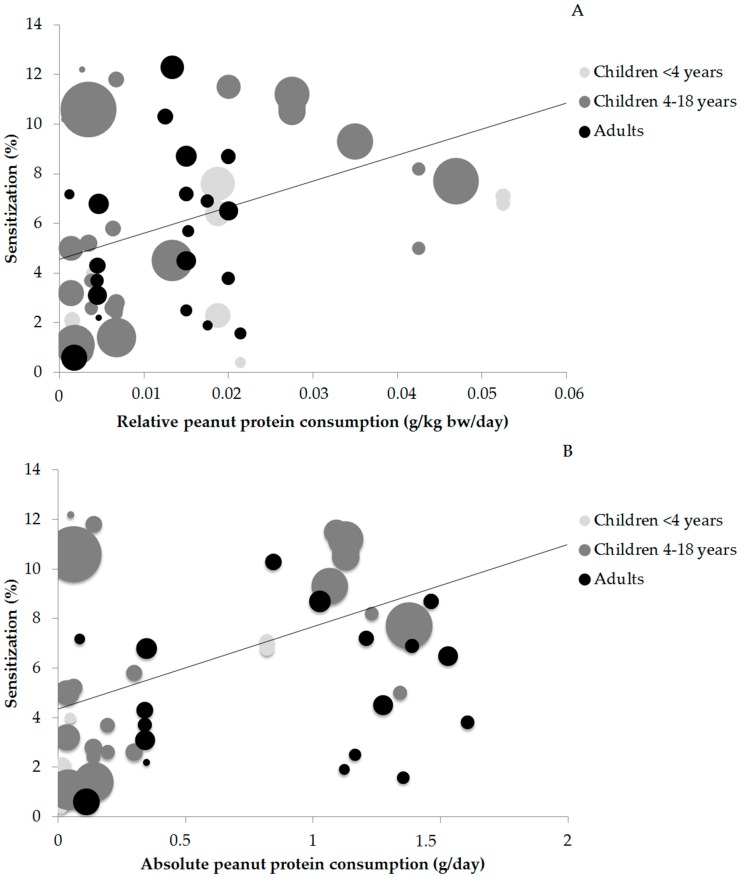
(**A**) Relation between relative peanut protein consumption (in g/kg bw/day) and reported peanut sensitization (%) in children <4 years; children 4–18 years; and adults. *r* values of respectively 0.505 (*p* > 0.05), 0.415 (*p* < 0.05), and 0.441 (*p* > 0.05) were found. The line represents the trend for all ages. (**B**) Relation between absolute peanut protein consumption (in g/day) and reported peanut sensitization in children <4 years; children 4–18 years; and adults. *r* values of respectively 0.493 (*p* < 0.05) in children 4–18 years and 0.461 (*p* > 0.05) were found in adults. The line represents the trend for all ages.

Sixty-one data points on the prevalence of peanut sensitization and peanut consumption were available for statistical analysis. The relation between peanut sensitization and peanut protein consumption are shown in Figure 2A (relative consumption) and Figure 2B (absolute consumption). The size of the circles indicates the size of the study population.

The *r* values between relative peanut protein consumption and peanut sensitization ranged from *r =* 0.415 (*p* < 0.05) in older children and *r =* 0.441 (*p* > 0.05) in adults to *r =* 0.505 (*p* > 0.05) in young children. The correlation in young children is not significant, which is most likely due to the low amount of data points (13). Evaluation of all ages together resulted in a low correlation value of *r =* 0.407 (*p* < 0.05) and the corresponding trend line is shown (Figure 2A).

It was not possible to determine the correlation between absolute peanut protein consumption and peanut sensitization in children below 4 years old due to the low number of publications (*n* = 7) and clustering of the data in a low and a high consumption cluster with no values in between. A low correlation was seen in children 4–18 years (*r =* 0.493) and in adults (*r =* 0.461). Assessment of all age groups together resulted in a low correlation of *r =* 0.468 and the corresponding trend line is shown in Figure 2B. Strong *r* values were not found, indicating that peanut protein consumption was not correlated to the prevalence of peanut sensitization. An overview of the *r* values for relative and absolute peanut protein consumption and sensitization can be found in Table 1.

### 3.4. Percentage of Consumers and Sensitization

**Figure 3:**
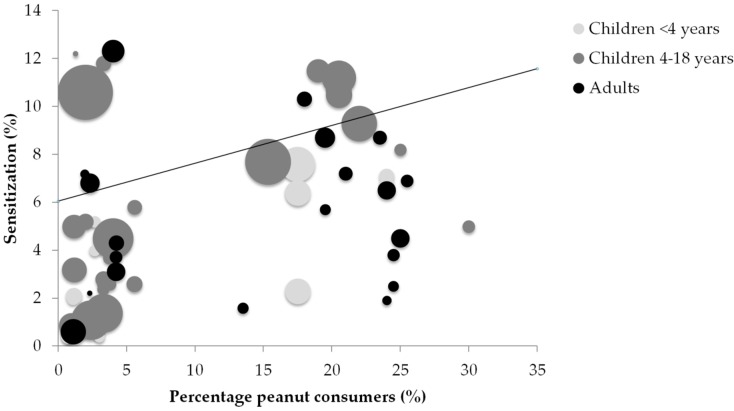
Relation between average peanut consumers (%) and reported peanut sensitization (%) in children <4 years; children 4–18 years; and adults, respectively. *r* values of respectively 0.673 (*p* < 0.05), 0.539 (*p* < 0.05), and 0.173 (*p* > 0.05) were found. The line represents the trend for all ages.

The results of the correlation between the percentage of peanut and peanut butter consumers in a country and peanut sensitization are shown in Figure 3. Correlation values ranged from *r =* 0.173 (*p* > 0.05) in adults and *r =* 0.539 (*p* < 0.05) in children 4–18 years to *r =* 0.673 (*p* < 0.05) in children <4 years. Inclusion of all ages together resulted in a nonsignificant negligible correlation of *r =* 0.243 (*p* > 0.05), indicating no correlation between the percentage of peanut consumers in a country and the prevalence of peanut sensitization in that country. The *r* values are reported in Table 1.

### 3.5. Peanut Allergen Content and Peanut Allergen Sensitization

Table 2 shows the mean Ara h 1, Ara h 2, Ara h 3, Ara h 6, Ara h 7, and Ara h 8 content relative to the total peanut protein amount. Ara h 1, 2, 3, and 6 content was determined by sodium dodecyl sulfate–polyacrylamide gel electrophoresis (SDS-PAGE) and reversed phase high-performance liquid chromatography (RP-HPLC) in 20 peanut varieties by Koppelman et al. [25]. For Ara h 7 and 8 less quantitative data were available.

**Figure 4:**
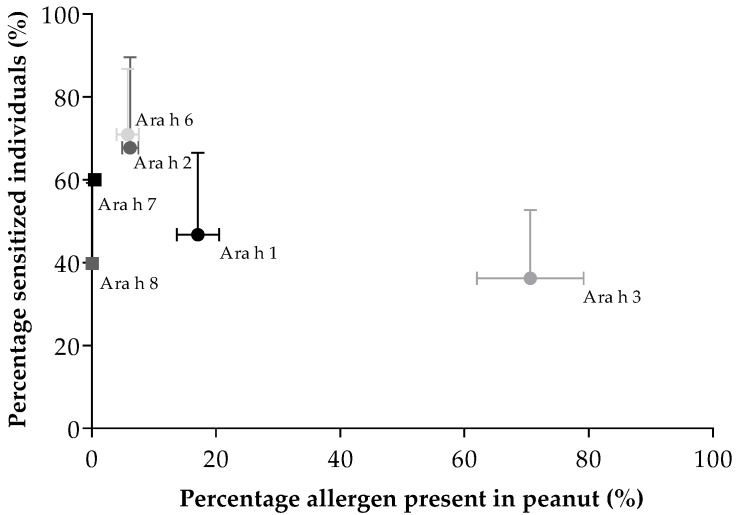
Relation between percentage of peanut sensitized individuals and percentage of peanut allergen in total peanut. The standard deviations are indicated by the horizontal (protein content) and vertical (allergen sensitization) error bars. A nonsignificant (*p* > 0.05) correlation (*r =* −0.257) was found between mean peanut content and mean allergen sensitization.

Articles investigating specific peanut allergen sensitization were retrieved and evaluated. A table with the included studies can be found in the Supplementary Materials (Table S5). Sensitization data for the individual peanut allergens were used to calculate the correlation with the individual allergen amount in peanut. For this purpose, articles investigating sensitization to Ara h 1 (*n* = 20), Ara h 2 (*n* = 21), Ara h 3 (*n* = 20), Ara h 6 (*n* = 8), Ara h 7 (*n* = 1), and Ara h 8 (*n* = 14) were used. Figure 4 shows the percentage of peanut allergic patients sensitized for peanut allergens and the percentage of these allergens present in the peanut. Sensitization to the 2S albumins Ara h 2 (70.72 ± 19.99%) and Ara h 6 (71.16 ± 15.66%) was most commonly seen, while the amount of these allergens present in peanut is rather low (6.2 ± 1.3% and 5.8 ± 1.8%, respectively). On the other hand, peanut contains only a small percentage of Ara h 7 (0.5%), whereas sensitization to Ara h 7 was high (60%). Furthermore, peanut contains a large amount of Ara h 3 (70.6 ± 8.6%) while sensitization to Ara h 3 is low (37.33 ± 16.25%). No significant (*p* > 0.05) correlation was found between mean peanut allergen content and mean prevalence of sensitization for peanut allergens.

### 3.6. Consumption and Sensitization Data for Other Legumes

**Figure 5:**
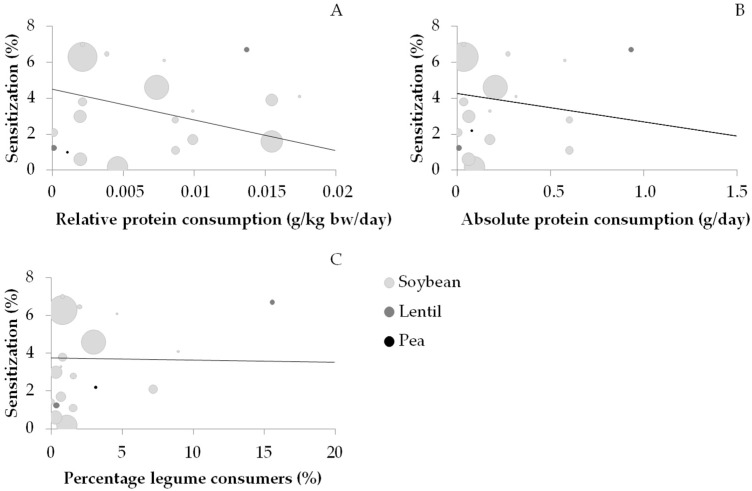
Sensitization data and data on relative consumption (**A**), absolute consumption (**B**), and the percentage of consumers (**C**) of soybean, lentil, and pea. The line represents the trend for soybean. Correlations between the prevalence of soybean sensitization and relative consumption (*r =* 0.352), absolute consumption (*r =* 0.217), and percentage of consumers (*r =* 0.007) were nonsignificant (*p* > 0.05).

Consumption and sensitization data were available for soybean (*n* = 17), lupin (*n* = 4), lentil (*n* = 2), and pea (*n* = 1). Lupin was excluded due to the lack of consumption data. Figure 5 shows the correlation between the prevalence of soybean, lentil, and pea sensitization and the relative consumption (Figure 5A), absolute consumption (Figure 5B), and percentage of consumers (Figure 5C). WLS linear regression was calculated for soybean but not for lentil and pea, on account of the low number of data points; however, these points are visualized in Figure 5. No significant correlation (*p* > 0.05) was found between the prevalence of soybean sensitization and the relative consumption (0.352), absolute consumption (0.217), and the percentage of consumers (0.007).

## 4. Discussion

To the best of our knowledge, this is the first investigation that systematically evaluates the association between the reported prevalence of legume sensitization in the general population from scientific publications and legume protein consumption using consumption surveys. We showed that, when all age groups are included, relative (g/kg bw/day) and absolute (g/kg/day) peanut protein consumption, as well as the percentage of peanut consumers, resulted in a low correlation with the prevalence of peanut sensitization in a country. No correlation was found between the prevalence of sensitization and consumption of soybean, and no conclusions could be drawn on pea, lupine, and lentil due to the limited number of data points. Furthermore, no significant correlation was found between peanut allergen content and sensitization to these allergens.

Studies investigating the relationship between peanut consumption and the prevalence of peanut sensitization are scarce. André et al. investigated in a French study population of 580 patients with reactions to foods over a period of 9 years the relationship between sensitization to food products and the consumption of these food products. Data on consumption were obtained from the French Institut National de la Statistique et des Etudes Economiques. André et al. concluded that despite the increased peanut consumption, there was a stable frequency of peanut sensitization (37%). Although the study of André et al. was limited to one country and an atopic study population, the results are in line with our results showing that there is no strong correlation between the amount of peanut consumption and peanut sensitization. On the contrary, André et al. found that for other foods, such as rice and wheat, increased sensitization did follow increased consumption. Additionally, a protective effect of dairy produce consumption in regards to sensitization to milk was reported.

The absolute and relative consumption values do not give a complete overview of the consumption behavior of a country. There can be a substantial percentage of peanut consumers, although consumption is low, resulting in proportionate low consumption. Therefore, the relationship between the percentage of peanut consumers and the prevalence of peanut sensitization was investigated, which resulted in no correlation between the variables. In addition, the frequency of consumption would be interesting to investigate, however, this information was not available and could not be calculated based on the reported values in the consumption surveys. The frequency of consumption could be used to investigate the effect of repeated exposure and its influence on sensitization. Frequency of consumption and the risk of sensitization were investigated in China by Yang et al., who concluded that there was no association between frequency of consumption and sensitization [28]. Nonetheless, more research is warranted in the area of frequency of consumption and the development of food sensitization.

To investigate if peanut protein content, and thus exposure to the specific peanut allergens, was correlated to peanut allergen sensitization, we investigated peanut protein content and the prevalence of peanut allergen sensitization reported in literature. No correlation was found between the protein content and allergen sensitization in peanut. This indicates that other factors are more important in causing sensitization. One of the possible factors is processing. In China, peanuts are mainly consumed raw, boiled, or fried, which may reduce the allergenicity of important peanut allergens [29,30,31]. Indeed, lower sensitization rates to Ara h 1 (5.6%), Ara h 2 (11.1%), and Ara h 3 (5.6%) were found in Chinese patients compared to patients from Western countries, where peanuts are generally consumed roasted or dry-roasted [29,30,32,33]. Multiple studies have studied the effect of thermal processing on peanut protein allergenicity. Another explanation for differences in the prevalence of sensitization could be cross-reactivity. A high prevalence of sensitization to the Ara h 8, a homologue of the major birch pollen allergen Bet v 1, is seen in countries such as Finland (53–79%) and Sweden (70%) where birch tree pollen is common [13,34,35], whereas low Ara h 8 sensitization is seen in Mediterranean countries with less birch tree pollen, such as Italy (25%) [36]. The high percentage of Ara h 8 sensitization in Northern Europe can thus be explained by cross-reactivity between Ara h 8 and Bet v 1.

Since no correlation between consumption and the prevalence of sensitization to legumes was found, we argue that other routes of exposure (e.g., cutaneous and respiratory route) besides the oral route influence the prevalence of peanut (allergen) sensitization. Prior research by Lack et al. has established that infants using skin creams containing peanut oil are at greater risk of developing peanut allergy [37]. Moreover, patients with atopic dermatitis and filaggrin loss-of-function mutations are prone to a dose-dependent increase in sensitization with increasing peanut dust levels [38,39]. Another study by Fox et al. found that high levels of environmental exposure to peanut during infancy appeared to promote sensitization [40]. The results from these publications indicate the important role of cutaneous exposure and its association with allergy and sensitization. Furthermore, reactions to peanut can occur from the first known oral exposure, indicating that sensitization already has occurred by either respiratory or cutaneous routes [41,42,43,44].

Another factor that might be associated with the prevalence of sensitization is the timing of introduction. Early introduction is associated with the induction of tolerance as shown by Du Toit et al. in infants [45]. Additionally, maternal peanut consumption during breast-feeding and introduction of peanut in the diet of the child in the first year of life was associated with a reduced risk of peanut sensitization [46]. Nevertheless, the efficacy of early introduction depends on adherence and dose, based on a large study in the United Kingdom by Perkin et al. [47]. The dual-allergen exposure hypothesis explains that low-dose cutaneous exposure leads to food sensitization and that early consumption leads to oral tolerance [48,49]. Finally, individual factors such as host genotype and microbiota are important factors in the development of allergy [50,51,52].

Besides the aforementioned factors, intrinsic properties of proteins also play a role in the development of sensitization, since not all patients are sensitized to the same proteins. Glycosylation, processing, and resistance to denaturation and digestion are all characteristics of an allergen that might influence sensitization [53]. For example, when Ara h 1 is boiled, morphologically distinct aggregates are formed with lower allergenicity, compared to roasting, which increases the allergenic potential of Ara h 1 [54,55]. The immunogenicity of Ara h 6 is also enhanced by heat denaturation [56]. It must be noted that these studies all investigated the effect on IgE binding; however, processing might also influence sensitization as well. Furthermore, it has been described that the conformational stability and resistance to digestion of Ara h 1 and Ara h 3 is lower compared to Ara h 2 and Ara h 6, which might explain why sensitization to Ara h 2 and Ara h 6 occurs more often compared to sensitization to Ara h 1 and Ara h 3 [57]. 

This study has some limitations. For most legumes, not enough sensitization and consumption data were available to be statistically evaluated. Consumption data from scientific publications were explored as an option, however, only a small number of publications were available and the studies were conducted in a study population or in countries without sensitization data [58,59,60]. The use of national import and export data was explored as another option but was hindered by other uses for legumes such as cattle feed. In the case of soybean, inconsistencies between consumption surveys in the EFSA were found. For example, a consumption survey in adolescents in Denmark only reported soy sauce consumption. In contrast, a consumption survey in the United Kingdom reported the consumption of seven soybean products (soy sauce, soya beans, textured soy protein, soya cheese, soya drink, soya yoghurt, and tofu). This might have influenced the consumption data and possibly the outcome of the analysis for soybean. Consumption of peanut butter and peanut oil was reported as one category in the GEMS/Food consumption database, which was the only source of information for Australia and China. Consumption values for these countries can be slightly different by reporting peanut oil and peanut butter as one category. However, peanut oil does not contain peanut protein according to the USDA Food Composition Databases. 

Various strengths of our study can be identified. One of the strengths of the study is the large number of articles included that reported on legume sensitization. Articles were selected based on an exhaustive search and systematically evaluated using a custom evaluation sheet. Legume consumption was assessed using reputable consumption surveys in multiple countries. Consumption was measured in relative and absolute values, which allowed for a more detailed evaluation of consumption behavior in a country. In addition, the percentage of consumers in a country helped to characterize consumption behavior and the effect on the prevalence of sensitization.

## 5. Conclusions

This study shows that the amount of peanut and soybean consumption and the percentage of consumers only plays a minor role in the prevalence of sensitization in a country. This indicates that other factors such as the intrinsic properties of the different proteins, processing, matrix, frequency, timing and route of exposure, and patient factors might play a more important role in relation to the prevalence of peanut sensitization than the amount of consumption.

## Figures and Tables

**Table 1:** Correlation (*r* values) for relative and absolute consumption with sensitization and percentage of peanut consumers with sensitization using WLS linear regression analysis.

Age Group	Relative Consumption and Sensitization (Figure 2A)	Absolute Consumption and Sensitization (Figure 2B)	Percentage Peanut Consumers and Sensitization (Figure 3)
Children <4 years	*r =* 0.505 (*p* > 0.05)	*-*	*r =* 0.673 (*p* < 0.05)
Children 4–18 years	*r =* 0.415 (*p* < 0.05)	*r =* 0.493 (*p* < 0.05)	*r =* 0.539 (*p* < 0.05)
Adults	*r =* 0.441 (*p* < 0.05)	*r =* 0.461 (*p* < 0.05)	*r =* 0.173 (*p* > 0.05)
All ages	*r =* 0.407 (*p* < 0.05)	*r =* 0.468 (*p* < 0.05)	*r =* 0.243 (*p* > 0.05)

**Table 2:** Percentage of total protein content of individual peanut allergens.

Protein	Reference	Content (± standard deviation) (%)
Ara h 1	Koppelman et al. [25]	17.1 (± 3.4)
Ara h 2	Koppelman et al. [25]	6.2 (± 1.3)
Ara h 3	Koppelman et al. [25]	70.6 (± 8.6)
Ara h 6	Koppelman et al. [25]	5.8 (± 1.8)
Ara h 7	Van Erp et al. [26]	0.5
Ara h 8	Lange et al. [27]	<0.1

## Supplementary Materials

The following are available online at http://www.mdpi.com/2072-6643/10/10/1545/s1. Table S1 contains details about the different allergens in legumes. Tables S2 and S3 detail the search queries in the different electronic bibliographic databases. Table S4 contains a table with the specifics of each included study investigating legume sensitization and the corresponding consumption values. The details of the included studies investigating component resolved diagnostics in peanut allergic patients can be found in Table S5.
