# Supplementary material for: Legume Protein Consumption and the Prevalence of Legume Sensitization
Source: Nutrients. 2018 Oct 19;10(10):1545. doi: 10.3390/nu10101545 (PMC6213573; doi:10.3390/nu10101545)
Supplement: Supplementary file 1 [file nutrients-10-01545-s001.pdf]

Table S1. Details of legume allergens (WHO/IUIS Allergen database (allergen.org)).

Species	Allergen	Protein family
Arachis hypogaea (Peanut)		
	Ara h 1	Cupin (Vicillin-type, 7S globulin)
	Ara h 2	Conglutin (2S albumin)
	Ara h 3	Cupin (Legumin-type, 11S globulin, Glycinin)
	Ara h 4	renamed to Ara h 3.02, number not available for future submissions
	Ara h 5	Profilin
	Ara h 6	Conglutin (2S albumin)
	Ara h 7	Conglutin (2S albumin)
	Ara h 8	Pathogenesis-related protein, PR-10, Bet v 1 family member
	Ara h 9	Nonspecific lipid-transfer protein type 1
	Ara h 10	16 kDa oleosin
	Ara h 11	14 kDa oleosin
	Ara h 12	Defensin
	Ara h 13	Defensin
	Ara h 14	Oleosin
	Ara h 15	Oleosin
	Ara h 16	nonspecific Lipid Transfer Protein 2
	Ara h 17	nonspecific Lipid Transfer Protein 1
Glycine max (Soybean)		
	Gly m 1	Hydrophobic protein from soybean
	Gly m 2	Defensin
	Gly m 3	Profilin
	Gly m 4	Pathogenesis-related protein, PR-10, Bet v 1 family member
	Gly m 5	Beta-conglycinin (vicilin, 7S globulin)
	Gly m 6	Glycinin (legumin, 11S globulin)
	Gly m 7	Seed biotinylated protein
	Gly m 8	2S albumin
Lens culinaris (Lentil)		
	Len c 1	Gamma-vicilin subunit
	Len c 2	Seed-specific biotinylated protein
	Len c 3	Nonspecific lipid transfer protein type 1
Lupinus albus (White lupin)		
	Lup an 5	Profilin
Lupinus angustifolius (narrow-leaved blue lupin)		
	Lup an 1	Conglutin beta (7S seed storage globulin, vicilin)
Phaseolus vulgaris (Green bean, French bean		
	Pha v 3	Nonspecific lipid transfer protein type 1
Pisum sativum (Pea)		
	Pis s 1	Vicilin
	Pis s 2	Convicilin
	Pis s 3	nsLTP

Vigna radiata (Mung bean)	
Vig r 1	Pathogenesis-related protein, PR-10, Bet v 1 family member
Vig r 2	8S Globulin (Vicilin)
Vig r 3	Renamed to Vig r 2.0201
Vig r 4	Seed albumin
Vig r 5	Identified as fragment of Vig r 2
Vig r 6	Cytokinin-specific binding protein (CSBP), Bet v 1 family member

Table S2. Search queries for articles reporting legume sensitization in different electronic bibliographic databases.

**Bibliographic database**	**Search query (performed on: 08-12-2017)**
Scopus	(Article title, Abstract, Keywords): Prevalence AND (Food hypersensitivity OR Food allergy) AND (Fabaceae OR Legume OR Pea OR Lentil OR Bean OR Peanut OR Soybean OR Lupin OR Chickpea)
Web of Science	(All databases) TOPIC: (Prevalence AND (Food hypersensitivity OR Food allergy) AND (Fabaceae OR Legume OR Pea OR Lentil OR Bean OR Peanut OR Soybean OR Lupin OR Chickpea))
Pubmed	(All Fields): Prevalence AND (Food hypersensitivity [Mesh] OR Food allergy) AND (Fabaceae [Mesh] OR Legume OR Pea OR Lentil OR Bean OR Peanut OR Soybean OR Lupin OR Chickpea)
EMbase	(Article title, Abstract, Author Keywords): Prevalence AND (Food AND hypersensitivity OR (Food AND allergy)) AND (Fabaceae OR Legume OR Pea OR Lentil OR Bean OR Peanut OR Soybean OR Lupin OR Chickpea)

Table S3. Search queries for peanut allergen sensitization in different electronic bibliographic databases.

**Bibliographic database**	**Search query (performed on: 27-04-2018)**
Scopus	(Article title, Abstract, Keywords): Sensitization AND "Ara h"
Web of Science	(All databases) TOPIC: Sensitization AND "Ara h"
Pubmed	(All Fields): (Sensitization AND "Ara h")
EMbase	(Article title, Abstract, Author Keywords): Sensitization AND "Ara h"

Table S4. Overview of articles (n = 42) investigating legume sensitization and relative and absolute peanut consumption values taken from EFSA, GEMS and WWEIA consumption surveys. Values are corrected for amount of peanut protein (25%).

**Authors**	**Nation**	**Study size**	**Reported age in years (mean or range)**	**Reported prevalence (%)**	**Peanut protein consumption (g/kg bw/day)**	**Peanut protein consumption (g/day)**	**Average percentage peanut consumers (%)**
Arshad et al. [1]	United Kingdom	978	4	1.1 Pn	0.007	0.14	3.3
Baatenburg de Jong et al. [2]	Netherlands	9044	0-18	7.7 Pn<br>4.6 S	0.047	1.38	15.3
Bähr et al. [3]	Germany	92	44	2.2 P<br>2.2 Pn<br>3.3 S<br>2.2 Lp	0.005	0.35	2.3
Björnsson et al. [4]	Sweden	1397	20-44	3.1 Pn	0.004	0.34	4.2
Branum et al. [5]	United States	5369	1-17	9.3 Pn	0.035	1.07	22.0
Bunyavanich et al. [6]	United States	616	8	5.0 Pn	0.043	1.34	30.0
Burney et al. [7]	Switzerland	485	38	5.04 Pn<br>5.07 Ln<br>4.61 S	n.a.*	n.a.	n.a.
	Spain	310	37.4	7.18 Pn	0.001	0.08	2.0

				6.71 Ln<br>6.47 S			
	Netherlands	476	36.5	1.58 Pn<br>1.24 Ln<br>1.45 S	0.021	1.35	13.5
	Poland	379	38.6	3.12 Pn<br>2.87 Ln<br>2.35 S	n.a.	n.a.	n.a.
	Bulgaria	113	37.6	1.79 Pn<br>4.48 Ln<br>1.79 S	n.a.	n.a.	n.a.
	Iceland	479	39.4	0.45 Pn<br>0.74 Ln<br>0.15 S	n.a.	n.a.	n.a.
Chen et al. [8]	China	477	0-1	0.4 Pn	0.021**	n.a.	3.0
Eller et al. [9]	Denmark	501	1-6	5.2 Pn	0.004	0.05	2.7
Ernst et al. [10]	Germany	13100	3-17	10.6 Pn<br>6.3 S	0.003	0.06	2.0
Gayraud et al.[11]	Belgium	2680	0-15	0.87 Pn<br>0.2 Lp	0.002	0.04	1.0
		2686	>15	0.6 Pn<br>0.6 Lp	0.002	0.11	1.1
Gislason et al. [12]	Iceland	570	33	0.8 Pn<br>0.2 S	n.a.	n.a.	n.a.
	Sweden	625	32.6	3.7 Pn<br>2.8 S	0.004	0.34	4.2
Grundy et al. [13]	United Kingdom	1246	3.2	3.3 Pn	0.006	0.02	1.1
Hourihane et al. [14]	United Kingdom	1072	4-5	2.8 Pn	0.007	0.14	3.3
Jøhnke et al. [15]	Denmark	497	1.5	4.0 Pn	0.004	0.05	2.7
Kim et al. [16]	China	2118	11-71	12.3 Pn<br>3.9 S	0.013***	n.a.	4.0
Krause et al. [17]	Denmark	1031	5-18	2.6 Pn<br>2.1 S	0.006	0.30	5.6
Liu et al. [18]	United States	909	1-5	7.1 Pn	0.053	0.82	24.0
		2869	6-19	10.7 Pn	0.028	1.13	20.5
		1672	20-39	8.7 Pn	0.015	1.03	19.5
		1361	40-59	6.5 Pn	0.020	1.53	24.0
		1392	>60	4.5 Pn	0.015	1.28	25.0
Mahesh et al. [19]	India	10931	34.3	8.78 Ln<br>9.24 S<br>8.73 Pn	n.a.	n.a.	n.a.
Martin et al. [20]	Australia	2739	1	2.3 Pn	0.019****	n.a.	17.5
Matricardi et al. [21]	Germany	273	2-10	7.0 S	n.a.	n.a.	n.a.
McGowan et al. [22]	United States	4995	6-19	11.2 Pn	0.028	1.13	20.5
		2901	6-19	10.5 Pn	0.028	1.13	20.5
Mortz et al. [23]	Denmark	862	14.1	5.8 Pn	0.006	0.30	5.6
Mustafayev et al.[24]	Turkey	6963	10.8	0.7 Pn	n.a.	n.a.	n.a.
Nicolaou et al. [25]	United Kingdom	933	8	11.8 Pn	0.007	0.14	3.3
Osborne et al. [26]	Australia	2757	1	6.4 Pn	0.019****	n.a.	17.5
Östblom et al. [27]	Sweden	2336	4	5.0 Pn<br>3.0 S	0.001	0.04	1.2
Pénard-Morand et al. [28]	France	6672	9-11	1.1 Pn	0.002	0.04	2.4
Pereira et al. [29]	United Kingdom	699	11	3.7 Pn	0.004	0.20	3.8
		649	15	2.6 Pn	0.004	0.20	3.8
Peters et al. [30]	Australia	5129	1	7.6 Pn	0.019****	n.a.	17.5
Rentzos et al. [31]	Sweden	944	18-75	2.8 Pn	0.004	0.34	4.2

				1.6 B<br>0.4 P<br>0.2 S			
Roberts et al. [32]	United Kingdom	6412	7	1.4 Pn<br>0.2 S	0.007	0.14	3.3
Ruokolainen et al. [33]	Finland	98	7-11	12.2 Pn<br>4.1 S	0.003	0.05	1.3
	Russia	82	7-11	1.2 Pn<br>0 S	n.a.	n.a.	n.a.
	Finland	98	15-20	10.2 Pn<br>6.1 S	0.001	0.03	0.7
	Russia	82	15-20	0 Pn<br>0 S	n.a.	n.a.	n.a.
Salo et al. [34]	United States	856	1-5	6.8 Pn	0.053	0.82	24.0
		580	6-9	8.2 Pn	0.043	1.23	25.0
		2277	10-19	11.5 Pn	0.020	1.09	19.0
		912	20-29	10.3 Pn	0.013	0.85	18.0
		756	30-39	7.2 Pn	0.015	1.21	21.0
		768	40-49	8.7 Pn	0.020	1.46	23.5
		588	50-59	3.8 Pn	0.020	1.61	24.5
		620	60-69	6.9 Pn	0.018	1.39	25.5
		444	70-79	2.5 Pn	0.015	1.17	24.5
		323	>80	1.9 Pn	0.018	1.12	24.0
Schäfer et al. [35]	Germany	1537	50	6.8 Pn<br>1.7 S	0.005	0.35	2.3
Schnabel et al. [36]	Germany	1082	2	2.1 Pn	0.002	0.02	1.2
		1082	6	5.2 Pn<br>3.8 S	0.003	0.06	2.0
Strinnholm et al. [37]	Sweden	2585	7-8	3.2 Pn<br>0.6 S	0.001	0.04	1.2
Sun et al. [38]	China	7085	9.6	4.5 Pn<br>1.6 S	0.013***	n.a.	4.0
Tariq et al. [39]	United Kingdom	1218	4	1.2 Pn	0.007	0.14	3.3
Venter et al. [40]	United Kingdom	700	6	2.6 Pn	0.007	0.14	3.3
Venter et al. [41]	United Kingdom	763	1	0.4 Pn	0.002	0.01	0.7
		658	2	3.3 Pn	0.001	0.02	1.1
		642	3	2.0 Pn	0.001	0.02	1.1
		588	10	2.4 Pn<br>0.68 Lp	0.007	0.14	3.3
Woods et al. [42]	Australia	457	39.2	5.7 Pn	0.015*****	n.a.	19.5

Pn = peanut; S = soybean; P = green pea; Lp = lupine; Ln = lentil; B = bean. *n.a. = no consumption values were available for this country. ** Consumption value from “Children” from the 2002 China Nutrition and Health Survey. *** Consumption value from “General population” from the 2002 China Nutrition and Health Survey. ****Consumption value from “Children 2-6-year-old” from the 2007 Australian National Children's Nutrition and Physical Activity Survey. *****Consumption value from “Children 2-16-year-old” from the 2007 Australian National Children's Nutrition and Physical Activity Survey.

Table S5. Articles (n = 21) investigating peanut allergen sensitization in peanut allergic children and adults.

Author	Reported prevalence of sensitization (%)					
	**Ara h 1**	**Ara h 2**	**Ara h 3**	**Ara h 6**	**Ara h 7**	**Ara h 8**
Agabriel et al. [43]	60.0	63.0	46.0	64.0		25.0
Blankestijn et al. [44]	43.0	65.0	38.0	60.0	60.0	
Uotila et al. [45]	43.0	57.0	36.0	60.0		53.0
Ma et al. [46]	5.6	11.0	5.6			22.2
Comberiati et al. [47]	8.3	58.3	16.7			25.0
Kukkonen et al. [48]	64.0	95.0	57.0	98.0		79.0

Bernard et al.[49]	56.0	77.0				
Ebisawa et al.[50]	38.0	88.0	42.0			42.0
Lopes de Oliveira et al. [51]	90.0	94.0	78.0			
Suratannon et al. [52]	26.3	68.4	21.1			15.8
Ballmer-Weber et al. [53]	44.0	56.0	25.0	50.0		34.0
Pedrosa et al.[54]	54.6	59.1	27.3	77.3		27.3
Ackerbauer et al. [55]	62.0	71.0	35.0	71.0		45.0
Bégin et al. [56]	63.0	95.0	43.0			
Arkwright et al. [57]	46.0	75.0	36.0			21.0
Pedrosa et al. [58]	60.0	72.7	43.6			
Movérare et al. [59]	52.7	56.8	44.6			70.3
Peeters et al. [60]	53.0	83.0	50.0	87.0		
Astier et al.[61]	40.0	100.0	27.0			
Klemans et al.[62]	25.6	43.0	16.3			59.3
Van Erp et al.[63]		33.7				38.6
Mean sensitization percentages	46.8	67.7	36.2	70.9	60.0	39.8

References

1. Arshad, S.H.; Tariq, S.M.; Matthews, S.; Hakim, E. Sensitization to common allergens and its association with allergic disorders at age 4 years: A whole population birth cohort study. *Pediatrics* **2001**, *108*, E33.
2. de Jong, A.B.; Dikkeschei, L.D.; Brand, P.L. Sensitization patterns to food and inhalant allergens in childhood: A comparison of non-sensitized, monosensitized, and polysensitized children. *Pediatr Allergy Immunol* **2011**, *22*, 166-171.
3. Bahr, M.; Fechner, A.; Kaatz, M.; Jahreis, G. Skin prick test reactivity to lupin in comparison to peanut, pea, and soybean in atopic and non-atopic german subjects: A preliminary cross-sectional study. *Immun Inflamm Dis* **2014**, *2*, 114-120.
4. Bjornsson, E.; Janson, C.; Plaschke, P.; Norrman, E.; Sjoberg, O. Prevalence of sensitization to food allergens in adult swedes. *Ann Allergy Asthma Immunol* **1996**, *77*, 327-332.
5. Branum, A.M.; Lukacs, S.L. Food allergy among children in the united states. *Pediatrics* **2009**, *124*, 1549-1555.
6. Bunyavanich, S.; Rifas-Shiman, S.L.; Platts-Mills, T.A.; Workman, L.; Sordillo, J.E.; Gillman, M.W.; Gold, D.R.; Litonjua, A.A. Peanut allergy prevalence among school-age children in a us cohort not selected for any disease. *J Allergy Clin Immunol* **2014**, *134*, 753-755.
7. Burney, P.G.; Potts, J.; Kummeling, I.; Mills, E.N.; Clausen, M.; Dubakiene, R.; Barreales, L.; Fernandez-Perez, C.; Fernandez-Rivas, M.; Le, T.M., *et al.* The prevalence and distribution of food sensitization in european adults. *Allergy* **2014**, *69*, 365-371.
8. Chen, J.; Hu, Y.; Allen, K.J.; Ho, M.H.; Li, H. The prevalence of food allergy in infants in chongqing, china. *Pediatr Allergy Immunol* **2011**, *22*, 356-360.
9. Eller, E.; Kjaer, H.F.; Host, A.; Andersen, K.E.; Bindslev-Jensen, C. Food allergy and food sensitization in early childhood: Results from the darc cohort. *Allergy* **2009**, *64*, 1023-1029.
10. Ernst, S.A.; Schmitz, R.; Thamm, M.; Ellert, U. Lower prevalence of atopic dermatitis and allergic sensitization among children and adolescents with a two-sided migrant background. *Int J Environ Res Public Health* **2016**, *13*.
11. Gayraud, J.; Mairesse, M.; Fontaine, J.F.; Thillay, A.; Leduc, V.; Rance, F.; Parisot, L.; Moneret-Vautrin, D.A. The prevalence of sensitization to lupin flour in france and belgium: A prospective study in 5,366 patients, by the allergy vigilance network. *Eur Ann Allergy Clin Immunol* **2009**, *41*, 17-22.
12. Gislason, D.; Bjornsson, E.; Gislason, T.; Janson, C.; Sjoberg, O.; Elfman, L.; Boman, G. Sensitization to airborne and food allergens in reykjavik (iceland) and uppsala (sweden) - a comparative study. *Allergy* **1999**, *54*, 1160-1167.
13. Grundy, J.; Matthews, S.; Bateman, B.; Dean, T.; Arshad, S.H. Rising prevalence of allergy to peanut in children: Data from 2 sequential cohorts. *J Allergy Clin Immunol* **2002**, *110*, 784-789.
14. Hourihane, J.O.; Aiken, R.; Briggs, R.; Gudgeon, L.A.; Grimshaw, K.E.; DunnGalvin, A.; Roberts, S.R. The impact of government advice to pregnant mothers regarding peanut avoidance on the prevalence of peanut allergy in united kingdom children at school entry. *J Allergy Clin Immunol* **2007**, *119*, 1197-1202.

15. Johnke, H.; Norberg, L.A.; Vach, W.; Host, A.; Andersen, K.E. Patterns of sensitization in infants and its relation to atopic dermatitis. *Pediatr Allergy Immunol* **2006**, *17*, 591-600.
16. Kim, J.S.; Ouyang, F.; Pongracic, J.A.; Fang, Y.; Wang, B.; Liu, X.; Xing, H.; Caruso, D.; Liu, X.; Zhang, S., *et al.* Dissociation between the prevalence of atopy and allergic disease in rural china among children and adults. *J Allergy Clin Immunol* **2008**, *122*, 929-935 e924.
17. Krause, T.G.; Koch, A.; Poulsen, L.K.; Kristensen, B.; Olsen, O.R.; Melbye, M. Atopic sensitization among children in an arctic environment. *Clin Exp Allergy* **2002**, *32*, 367-372.
18. Liu, A.H.; Jaramillo, R.; Sicherer, S.H.; Wood, R.A.; Bock, S.A.; Burks, A.W.; Massing, M.; Cohn, R.D.; Zeldin, D.C. National prevalence and risk factors for food allergy and relationship to asthma: Results from the national health and nutrition examination survey 2005-2006. *J Allergy Clin Immunol* **2010**, *126*, 798-806 e713.
19. Mahesh, P.A.; Wong, G.W.; Ogorodova, L.; Potts, J.; Leung, T.F.; Fedorova, O.; Holla, A.D.; Fernandez-Rivas, M.; Clare Mills, E.N.; Kummeling, I., *et al.* Prevalence of food sensitization and probable food allergy among adults in india: The europrevall inco study. *Allergy* **2016**, *71*, 1010-1019.
20. Martin, P.E.; Eckert, J.K.; Koplin, J.J.; Lowe, A.J.; Gurrin, L.C.; Dharmage, S.C.; Vuillermin, P.; Tang, M.L.; Ponsonby, A.L.; Matheson, M., *et al.* Which infants with eczema are at risk of food allergy? Results from a population-based cohort. *Clin Exp Allergy* **2015**, *45*, 255-264.
21. Matricardi, P.M.; Bockelbrink, A.; Beyer, K.; Keil, T.; Niggemann, B.; Gruber, C.; Wahn, U.; Lau, S. Primary versus secondary immunoglobulin e sensitization to soy and wheat in the multi-centre allergy study cohort. *Clin Exp Allergy* **2008**, *38*, 493-500.
22. McGowan, E.C.; Peng, R.D.; Salo, P.M.; Zeldin, D.C.; Keet, C.A. Changes in food-specific ige over time in the national health and nutrition examination survey (nhanes). *J Allergy Clin Immunol Pract* **2016**, *4*, 713-720.
23. Mortz, C.G.; Andersen, K.E.; Bindslev-Jensen, C. The prevalence of peanut sensitization and the association to pollen sensitization in a cohort of unselected adolescents--the odense adolescence cohort study on atopic diseases and dermatitis (toacs). *Pediatr Allergy Immunol* **2005**, *16*, 501-506.
24. Mustafayev, R.; Civelek, E.; Orhan, F.; Yuksel, H.; Boz, A.B.; Sekerel, B.E. Similar prevalence, different spectrum: Ige-mediated food allergy among turkish adolescents. *Allergol Immunopathol (Madr)* **2013**, *41*, 387-396.
25. Nicolaou, N.; Poorafshar, M.; Murray, C.; Simpson, A.; Winell, H.; Kerry, G.; Harlin, A.; Woodcock, A.; Ahlstedt, S.; Custovic, A. Allergy or tolerance in children sensitized to peanut: Prevalence and differentiation using component-resolved diagnostics. *J Allergy Clin Immunol* **2010**, *125*, 191-197 e191-113.
26. Osborne, N.J.; Koplin, J.J.; Martin, P.E.; Gurrin, L.C.; Lowe, A.J.; Matheson, M.C.; Ponsonby, A.L.; Wake, M.; Tang, M.L.; Dharmage, S.C., *et al.* Prevalence of challenge-proven ige-mediated food allergy using population-based sampling and predetermined challenge criteria in infants. *J Allergy Clin Immunol* **2011**, *127*, 668-676 e661-662.
27. Ostblom, E.; Lilja, G.; Ahlstedt, S.; van Hage, M.; Wickman, M. Patterns of quantitative food-specific ige-antibodies and reported food hypersensitivity in 4-year-old children. *Allergy* **2008**, *63*, 418-424.
28. Penard-Morand, C.; Raherison, C.; Kopferschmitt, C.; Caillaud, D.; Lavaud, F.; Charpin, D.; Bousquet, J.; Annesi-Maesano, I. Prevalence of food allergy and its relationship to asthma and allergic rhinitis in schoolchildren. *Allergy* **2005**, *60*, 1165-1171.
29. Pereira, B.; Venter, C.; Grundy, J.; Clayton, C.B.; Arshad, S.H.; Dean, T. Prevalence of sensitization to food allergens, reported adverse reaction to foods, food avoidance, and food hypersensitivity among teenagers. *J Allergy Clin Immunol* **2005**, *116*, 884-892.
30. Peters, R.L.; Koplin, J.J.; Gurrin, L.C.; Dharmage, S.C.; Wake, M.; Ponsonby, A.L.; Tang, M.L.K.; Lowe, A.J.; Matheson, M.; Dwyer, T., *et al.* The prevalence of food allergy and other allergic diseases in early childhood in a population-based study: Healthnuts age 4-year follow-up. *J Allergy Clin Immunol* **2017**, *140*, 145-153 e148.
31. Rentzos, G.; Johanson, L.; Sjolander, S.; Telemo, E.; Ekerljung, L. Self-reported adverse reactions and ige sensitization to common foods in adults with asthma. *Clin Transl Allergy* **2015**, *5*, 25.
32. Roberts, G.; Peckitt, C.; Northstone, K.; Strachan, D.; Lack, G.; Henderson, J.; Golding, J.; Team, A.S. Relationship between aeroallergen and food allergen sensitization in childhood. *Clin Exp Allergy* **2005**, *35*, 933-940.

33. Ruokolainen, L.; Paalanen, L.; Karkman, A.; Laatikainen, T.; von Hertzen, L.; Vlasoff, T.; Markelova, O.; Masyuk, V.; Auvinen, P.; Paulin, L., *et al.* Significant disparities in allergy prevalence and microbiota between the young people in finnish and russian karelia. *Clin Exp Allergy* **2017**, *47*, 665-674.
34. Salo, P.M.; Arbes, S.J., Jr.; Jaramillo, R.; Calatroni, A.; Weir, C.H.; Sever, M.L.; Hoppin, J.A.; Rose, K.M.; Liu, A.H.; Gergen, P.J., *et al.* Prevalence of allergic sensitization in the united states: Results from the national health and nutrition examination survey (nhanes) 2005-2006. *J Allergy Clin Immunol* **2014**, *134*, 350-359.
35. Schafer, T.; Bohler, E.; Ruhdorfer, S.; Weigl, L.; Wessner, D.; Heinrich, J.; Filipiak, B.; Wichmann, H.E.; Ring, J. Epidemiology of food allergy/food intolerance in adults: Associations with other manifestations of atopy. *Allergy* **2001**, *56*, 1172-1179.
36. Schnabel, E.; Sausenthaler, S.; Schaaf, B.; Schafer, T.; Lehmann, I.; Behrendt, H.; Herbarth, O.; Borte, M.; Kramer, U.; von Berg, A., *et al.* Prospective association between food sensitization and food allergy: Results of the lisa birth cohort study. *Clin Exp Allergy* **2010**, *40*, 450-457.
37. Strinnholm, A.; Winberg, A.; West, C.; Hedman, L.; Ronmark, E. Food hypersensitivity is common in swedish schoolchildren, especially oral reactions to fruit and gastrointestinal reactions to milk. *Acta Paediatr* **2014**, *103*, 1290-1296.
38. Sun, B.Q.; Chen, D.H.; Zheng, P.Y.; Huang, H.M.; Luo, W.T.; Zeng, G.Q.; Zhang, X.W. Allergy-related evidences in relation to serum ige: Data from the china state key laboratory of respiratory disease, 2008-2013. *Biomed Environ Sci* **2014**, *27*, 495-505.
39. Tariq, S.M.; Stevens, M.; Matthews, S.; Ridout, S.; Twiselton, R.; Hide, D.W. Cohort study of peanut and tree nut sensitisation by age of 4 years. *BMJ* **1996**, *313*, 514-517.
40. Venter, C.; Pereira, B.; Grundy, J.; Clayton, C.B.; Arshad, S.H.; Dean, T. Prevalence of sensitization reported and objectively assessed food hypersensitivity amongst six-year-old children: A population-based study. *Pediatr Allergy Immunol* **2006**, *17*, 356-363.
41. Venter, C.; Maslin, K.; Patil, V.; Kurukulaaratchy, R.; Grundy, J.; Glasbey, G.; Twiselton, R.; Dean, T.; Arshad, S.H. The prevalence, natural history and time trends of peanut allergy over the first 10 years of life in two cohorts born in the same geographical location 12 years apart. *Pediatr Allergy Immunol* **2016**, *27*, 804-811.
42. Woods, R.K.; Stoney, R.M.; Raven, J.; Walters, E.H.; Abramson, M.; Thien, F.C. Reported adverse food reactions overestimate true food allergy in the community. *Eur J Clin Nutr* **2002**, *56*, 31-36.
43. Agabriel, C.; Ghazouani, O.; Birnbaum, J.; Liabeuf, V.; Porri, F.; Gouitaa, M.; Cleach, I.; Grob, J.J.; Bongrand, P.; Sarles, J., *et al.* Ara h 2 and ara h 6 sensitization predicts peanut allergy in mediterranean pediatric patients. *Pediatr Allergy Immunol* **2014**, *25*, 662-667.
44. Blankestijn, M.A.; Otten, H.G.; Suer, W.; Weimann, A.; Knol, E.F.; Knulst, A.C. Specific ige to peanut 2s albumin ara h 7 has a discriminative ability comparable to ara h 2 and 6. *Clin Exp Allergy* **2018**, *48*, 60-65.
45. Uotila, R.; Kukkonen, A.K.; Blom, W.M.; Remington, B.; Westerhout, J.; Pelkonen, A.S.; Makela, M.J. Component-resolved diagnostics demonstrates that most peanut-allergic individuals could potentially introduce tree nuts to their diet. *Clin Exp Allergy* **2018**, *48*, 712-721.
46. Ma, S.; Nie, L.; Li, H.; Wang, R.; Yin, J. Component-resolved diagnosis of peanut allergy and its possible origins of sensitization in china. *Int Arch Allergy Immunol* **2016**, *169*, 241-248.
47. Comberiati, P.; Colavita, L.; Minniti, F.; Paiola, G.; Capristo, C.; Incorvaia, C.; Peroni, D.G. Utility of specific ige to ara h 2 in italian allergic and tolerant children sensitized to peanut. *Int J Mol Cell Med* **2016**, *5*, 160-166.
48. Kukkonen, A.K.; Pelkonen, A.S.; Makinen-Kiljunen, S.; Voutilainen, H.; Makela, M.J. Ara h 2 and ara 6 are the best predictors of severe peanut allergy: A double-blind placebo-controlled study. *Allergy* **2015**, *70*, 1239-1245.
49. Bernard, H.; Paty, E.; Mondoulet, L.; Burks, A.W.; Bannon, G.A.; Wal, J.M.; Scheinmann, P. Serological characteristics of peanut allergy in children. *Allergy* **2003**, *58*, 1285-1292.
50. Ebisawa, M.; Moverare, R.; Sato, S.; Maruyama, N.; Borres, M.P.; Komata, T. Measurement of ara h 1-, 2-, and 3-specific ige antibodies is useful in diagnosis of peanut allergy in japanese children. *Pediatr Allergy Immunol* **2012**, *23*, 573-581.
51. Lopes de Oliveira, L.C.; Aderhold, M.; Brill, M.; Schulz, G.; Rolinck-Werninghaus, C.; Clare Mills, E.N.; Niggemann, B.; Naspitz, C.K.; Wahn, U.; Beyer, K. The value of specific ige to peanut and its component ara h 2 in the diagnosis of peanut allergy. *J Allergy Clin Immunol Pract* **2013**, *1*, 394-398.

52. Suratannon, N.; Ngamphaiboon, J.; Wongpiyabovorn, J.; Puripokai, P.; Chatchatee, P. Component-resolved diagnostics for the evaluation of peanut allergy in a low-prevalence area. *Pediatr Allergy Immunol* **2013**, *24*, 665-670.
53. Ballmer-Weber, B.K.; Lidholm, J.; Fernandez-Rivas, M.; Seneviratne, S.; Hanschmann, K.M.; Vogel, L.; Bures, P.; Fritsche, P.; Summers, C.; Knulst, A.C., *et al.* Ige recognition patterns in peanut allergy are age dependent: Perspectives of the europrevall study. *Allergy* **2015**, *70*, 391-407.
54. Pedrosa, M.; Boyano-Martinez, T.; Garcia-Ara, C.; Caballero, T.; Quirce, S. Utility of specific ige to ara h 6 in peanut allergy diagnosis. *Ann Allergy Asthma Immunol* **2015**, *115*, 108-112.
55. Ackerbauer, D.; Bublin, M.; Radauer, C.; Varga, E.M.; Hafner, C.; Ebner, C.; Szepfalusi, Z.; Froschl, R.; Hoffmann-Sommergruber, K.; Eiwegger, T., *et al.* Component-resolved ige profiles in austrian patients with a convincing history of peanut allergy. *Int Arch Allergy Immunol* **2015**, *166*, 13-24.
56. Begin, P.; Vitte, J.; Paradis, L.; Paradis, J.; Bongrand, P.; Chanez, P.; Des Roches, A. Long-term prognostic value of component-resolved diagnosis in infants and toddlers with peanut allergy. *Pediatr Allergy Immunol* **2014**, *25*, 506-508.
57. Arkwright, P.D.; Summers, C.W.; Riley, B.J.; Alsediq, N.; Pumphrey, R.S. Ige sensitization to the nonspecific lipid-transfer protein ara h 9 and peanut-associated bronchospasm. *Biomed Res Int* **2013**, *2013*, 746507.
58. Pedrosa, M.; Boyano-Martinez, T.; Garcia-Ara, M.C.; Caballero, T.; Quirce, S. Peanut seed storage proteins are responsible for clinical reactivity in spanish peanut-allergic children. *Pediatr Allergy Immunol* **2012**, *23*, 654-659.
59. Moverare, R.; Ahlstedt, S.; Bengtsson, U.; Borres, M.P.; van Hage, M.; Poorafshar, M.; Sjolander, S.; Akerstrom, J.; van Odijk, J. Evaluation of ige antibodies to recombinant peanut allergens in patients with reported reactions to peanut. *Int Arch Allergy Immunol* **2011**, *156*, 282-290.
60. Peeters, K.A.; Koppelman, S.J.; van Hoffen, E.; van der Tas, C.W.; den Hartog Jager, C.F.; Penninks, A.H.; Hefle, S.L.; Bruijnzeel-Koomen, C.A.; Knol, E.F.; Knulst, A.C. Does skin prick test reactivity to purified allergens correlate with clinical severity of peanut allergy? *Clin Exp Allergy* **2007**, *37*, 108-115.
61. Astier, C.; Morisset, M.; Roitel, O.; Codreanu, F.; Jacquenet, S.; Franck, P.; Ogier, V.; Petit, N.; Proust, B.; Moneret-Vautrin, D.A., *et al.* Predictive value of skin prick tests using recombinant allergens for diagnosis of peanut allergy. *J Allergy Clin Immunol* **2006**, *118*, 250-256.
62. Klemans, R.J.; Broekman, H.C.; Knol, E.F.; Bruijnzeel-Koomen, C.A.; Otten, H.G.; Pasmans, S.G.; Knulst, A.C. Ara h 2 is the best predictor for peanut allergy in adults. *J Allergy Clin Immunol Pract* **2013**, *1*, 632-638 e631.
63. van Erp, F.C.; Knol, E.F.; Pontoppidan, B.; Meijer, Y.; van der Ent, C.K.; Knulst, A.C. The ige and basophil responses to ara h 2 and ara h 6 are good predictors of peanut allergy in children. *J Allergy Clin Immunol* **2017**, *139*, 358-360 e358.
